# Supplementary material for: Responses of soil labile organic carbon stocks and the carbon pool management index to different vegetation restoration types in the Danxia landform region of southwest China
Source: PLoS One. 2025 Feb 25;20(2):e0318195. doi: 10.1371/journal.pone.0318195 (PMC11856273; doi:10.1371/journal.pone.0318195)
Supplement: S1 Text — S1 Table. Mean (±SE, n = 3) soil basic physical and chemical properties in the five vegetation types. S2 Table.Two-way ANOVA of the effects of different vegetation types (VT) and soil depths (SD) on SOC and LOCFs. S3 Table.The proportion of LOCFs (DOC, MBC, and EOC) in total SOC (%) of the five vegetation types. S4 Table. LOCFs (DOC, MBC, and EOC) stocks in the different vegetation types. S5 Table. SOC pool indexes (L, LI, CPI, and CPMI) of the different vegetation types. S1 Fig. SOC concentration (a) and stocks (b) in each soil layer of the different vegetation types. S2 Fig. LOCF concentrations of the different vegetation types. a) DOC, b) MBC, and c) EOC. S3 Fig. Redundancy analysis of soil carbon fractions and physicochemical properties. (ZIP) [file pone.0318195.s001.zip › Supporting information/Raw data.docx]

**S1 Table. Mean (±SE, n=3) soil basic physical and chemical properties in the five vegetation types.**

| pH值 |  | Soil depth (cm) | Repeat1 | Repeat2 | Repeat3 | Average | STDE |
| --- | --- | --- | --- | --- | --- | --- | --- |
|  | SH | 0-10 | 7.51 | 7.73 | 7.62 | 7.62 | 0.11 |
|  |  | 10-20 | 8.21 | 8.14 | 8.18 | 8.18 | 0.04 |
|  |  | 20-30 | 8.15 | 8.07 | 8.11 | 8.11 | 0.04 |
|  |  | Average | 7.96 | 7.98 | 7.97 | 7.97 | 0.06 |
|  |  |  |  |  |  |  |  |
|  | BF | 0-10 | 5.40 | 5.57 | 5.49 | 5.49 | 0.09 |
|  |  | 10-20 | 5.82 | 5.99 | 5.91 | 5.91 | 0.09 |
|  |  | 20-30 | 5.88 | 5.88 | 5.88 | 5.88 | 0.00 |
|  |  | Average | 5.70 | 5.81 | 5.76 | 5.76 | 0.06 |
|  |  |  |  |  |  |  |  |
|  | CFF | 0-10 | 4.94 | 6.05 | 5.95 | 5.65 | 0.61 |
|  |  | 10-20 | 5.96 | 5.26 | 4.83 | 5.35 | 0.57 |
|  |  | 20-30 | 5.40 | 5.80 | 4.78 | 5.33 | 0.51 |
|  |  | Average | 5.43 | 5.70 | 5.19 | 5.44 | 0.57 |
|  |  |  |  |  |  |  |  |
|  | MCBF | 0-10 | 5.23 | 4.82 | 4.46 | 4.84 | 0.39 |
|  |  | 10-20 | 4.86 | 5.37 | 4.71 | 4.98 | 0.35 |
|  |  | 20-30 | 4.71 | 4.79 | 5.24 | 4.91 | 0.29 |
|  |  | Average | 4.93 | 4.99 | 4.80 | 4.91 | 0.34 |
|  |  |  |  |  |  |  |  |
|  | EBF | 0-10 | 4.64 | 4.35 | 4.53 | 4.51 | 0.15 |
|  |  | 10-20 | 4.87 | 5.08 | 5.14 | 5.03 | 0.14 |
|  |  | 20-30 | 4.96 | 5.35 | 5.32 | 5.21 | 0.22 |
|  |  | Average | 4.82 | 4.93 | 5.00 | 4.92 | 0.17 |

| Moisture | Soil depth (cm) | Repeat1 | Repeat2 | Repeat3 | Average | STDE |
| --- | --- | --- | --- | --- | --- | --- |
| SH | 0-10 | 23.24 | 20.22 | 21.73 | 21.73 | 1.51 |
|  | 10-20 | 15.92 | 17.26 | 16.59 | 16.59 | 0.67 |
|  | 20-30 | 16.28 | 12.74 | 14.51 | 14.51 | 1.77 |
|  | Average | 18.48 | 16.74 | 17.61 | 17.61 | 1.32 |
|  |  |  |  |  |  |  |
| BF | 0-10 | 27.54 | 31.46 | 29.50 | 29.50 | 1.96 |
|  | 10-20 | 23.53 | 20.63 | 22.08 | 22.08 | 1.45 |
|  | 20-30 | 21.66 | 20.33 | 21.00 | 21.00 | 0.67 |
|  | Average | 24.24 | 24.14 | 24.19 | 24.19 | 1.36 |
|  |  |  |  |  |  |  |
| CFF | 0-10 | 33.75 | 24.45 | 23.18 | 27.13 | 5.77 |
|  | 10-20 | 23.27 | 15.42 | 27.85 | 22.18 | 6.28 |
|  | 20-30 | 14.84 | 18.62 | 20.77 | 18.08 | 3.00 |
|  | Average | 23.96 | 19.50 | 23.93 | 22.46 | 5.02 |
|  |  |  |  |  |  |  |
| MBCF | 0-10 | 17.93 | 21.83 | 25.43 | 21.73 | 3.75 |
|  | 10-20 | 17.72 | 15.69 | 14.75 | 16.05 | 1.52 |
|  | 20-30 | 14.79 | 14.84 | 13.91 | 14.51 | 0.52 |
|  | Average | 16.81 | 17.45 | 18.03 | 17.43 | 1.93 |
|  |  |  |  |  |  |  |
| EBF | 0-10 | 37.45 | 32.10 | 37.05 | 35.53 | 2.98 |
|  | 10-20 | 24.91 | 24.05 | 22.24 | 23.73 | 1.37 |
|  | 20-30 | 27.06 | 23.01 | 20.14 | 23.41 | 3.47 |
|  | Average | 29.81 | 26.39 | 26.48 | 27.56 | 2.61 |

| BD（g.cm3) | Soil depth (cm) | Repeat1 | Repeat2 | Repeat3 | Average | STDE |
| --- | --- | --- | --- | --- | --- | --- |
| SH | 0-10 | 1.78 | 2.04 | 1.91 | 1.91 | 0.13 |
|  | 10-20 | 2.05 | 1.85 | 1.95 | 1.95 | 0.10 |
|  | 20-30 | 1.68 | 1.82 | 1.75 | 1.75 | 0.07 |
|  | Average | 1.84 | 1.90 | 1.87 | 1.87 | 0.10 |
| BF | 0-10 | 1.33 | 1.25 | 1.29 | 1.29 | 0.04 |
|  | 10-20 | 1.42 | 1.58 | 1.50 | 1.50 | 0.08 |
|  | 20-30 | 1.84 | 1.43 | 1.64 | 1.64 | 0.20 |
|  | Average | 1.53 | 1.42 | 1.48 | 1.48 | 0.11 |
| CFF | 0-10 | 1.59 | 1.47 | 1.75 | 1.60 | 0.14 |
|  | 10-20 | 1.41 | 1.68 | 1.54 | 1.54 | 0.14 |
|  | 20-30 | 1.84 | 1.30 | 1.68 | 1.61 | 0.28 |
|  | Average | 1.61 | 1.48 | 1.66 | 1.58 | 0.18 |
| MCBF | 0-10 | 2.17 | 1.96 | 1.56 | 1.90 | 0.31 |
|  | 10-20 | 2.07 | 2.07 | 1.56 | 1.90 | 0.29 |
|  | 20-30 | 1.91 | 1.81 | 1.93 | 1.88 | 0.06 |
|  | Average | 2.05 | 1.95 | 1.68 | 1.89 | 0.22 |
| EBF | 0-10 | 1.42 | 1.44 | 1.67 | 1.51 | 0.14 |
|  | 10-20 | 1.60 | 1.88 | 1.92 | 1.80 | 0.17 |
|  | 20-30 | 2.01 | 1.96 | 2.14 | 2.04 | 0.09 |
|  | Average | 1.68 | 1.76 | 1.91 | 1.78 | 0.14 |

| TN(g/kg) | Soil depth (cm) | Repeat1 | Repeat2 | Repeat3 | Average | STDE |
| --- | --- | --- | --- | --- | --- | --- |
| SH | 0-10 | 1.57 | 1.15 | 1.36 | 1.36 | 0.21 |
|  | 10-20 | 0.63 | 0.75 | 0.69 | 0.69 | 0.06 |
|  | 20-30 | 0.60 | 0.61 | 0.61 | 0.61 | 0.01 |
|  | Average | 0.93 | 0.84 | 0.89 |  |  |
| BF | 0-10 | 3.07 | 3.57 | 3.32 | 3.32 | 0.25 |
|  | 10-20 | 1.88 | 1.63 | 1.75 | 1.75 | 0.13 |
|  | 20-30 | 1.21 | 1.89 | 1.55 | 1.55 | 0.34 |
|  | Average | 2.05 | 2.36 | 2.21 |  |  |
| CFF | 0-10 | 2.15 | 1.12 | 1.07 | 1.45 | 0.61 |
|  | 10-20 | 0.97 | 0.61 | 0.79 | 0.79 | 0.18 |
|  | 20-30 | 0.59 | 0.80 | 0.69 | 0.69 | 0.11 |
|  | Average | 1.24 | 0.84 | 0.85 |  |  |
| MCBF | 0-10 | 0.79 | 0.87 | 1.01 | 0.89 | 0.12 |
|  | 10-20 | 0.38 | 0.39 | 0.32 | 0.36 | 0.04 |
|  | 20-30 | 0.27 | 0.30 | 0.25 | 0.27 | 0.03 |
|  | Average | 0.48 | 0.52 | 0.53 |  |  |
| EBF | 0-10 | 2.81 | 1.64 | 1.65 | 2.03 | 0.68 |
|  | 10-20 | 1.06 | 0.96 | 0.73 | 0.92 | 0.17 |
|  | 20-30 | 0.90 | 0.75 | 0.55 | 0.74 | 0.18 |
|  | Average | 1.59 | 1.12 | 0.98 |  |  |
|  |  |  |  |  |  |  |
| TP(g/kg) |  |  |  |  |  |  |
|  | Soil depth (cm) | Repeat1 | Repeat2 | Repeat3 | Average | STDE |
| SH | 0-10 | 0.42 | 0.41 | 0.42 | 0.42 | 0.00 |
|  | 10-20 | 0.42 | 0.40 | 0.41 | 0.41 | 0.01 |
|  | 20-30 | 0.40 | 0.39 | 0.39 | 0.39 | 0.00 |
|  | Average | 0.41 | 0.40 | 0.41 | 0.41 | 0.00 |
| BF | 0-10 | 0.62 | 0.76 | 0.89 | 0.76 | 0.13 |
|  | 10-20 | 0.67 | 0.57 | 0.62 | 0.62 | 0.05 |
|  | 20-30 | 0.39 | 0.65 | 0.52 | 0.52 | 0.13 |
|  | Average | 0.56 | 0.66 | 0.68 | 0.63 | 0.10 |
| CFF | 0-10 | 0.27 | 0.26 | 0.25 | 0.26 | 0.01 |
|  | 10-20 | 0.27 | 0.18 | 0.27 | 0.24 | 0.05 |
|  | 20-30 | 0.19 | 0.21 | 0.25 | 0.22 | 0.03 |
|  | Average | 0.25 | 0.22 | 0.26 | 0.24 | 0.03 |
| MCBF | 0-10 | 0.22 | 0.22 | 0.20 | 0.21 | 0.01 |
|  | 10-20 | 0.18 | 0.18 | 0.14 | 0.17 | 0.02 |
|  | 20-30 | 0.14 | 0.14 | 0.13 | 0.14 | 0.01 |
|  | Average | 0.18 | 0.18 | 0.16 | 0.17 | 0.01 |
| EBF | 0-10 | 0.30 | 0.23 | 0.22 | 0.25 | 0.05 |
|  | 10-20 | 0.23 | 0.19 | 0.20 | 0.21 | 0.02 |
|  | 20-30 | 0.21 | 0.20 | 0.18 | 0.20 | 0.01 |
|  | Average | 0.25 | 0.21 | 0.20 | 0.22 | 0.03 |

**S2 Table.Two-way ANOVA of the effects of different vegetation types (VT) and soil depths (SD) on SOC and LOCFs.**

| SOC(g/kg) |  |  |  |  |  |  |
| --- | --- | --- | --- | --- | --- | --- |
|  | Soil depth (cm) | Repeat1 | Repeat2 | Repeat3 | Average | STDE |
| SH | 0-10 | 17.52 | 12.98 | 15.25 | 15.25 | 2.27 |
|  | 10-20 | 5.60 | 6.28 | 5.94 | 5.94 | 0.34 |
|  | 20-30 | 4.80 | 4.30 | 4.55 | 4.55 | 0.25 |
|  | Average | 9.31 | 7.85 | 8.58 |  |  |
| BF | 0-10 | 31.83 | 35.10 | 33.47 | 33.47 | 1.63 |
|  | 10-20 | 19.82 | 15.00 | 17.41 | 17.41 | 2.41 |
|  | 20-30 | 13.59 | 15.49 | 14.54 | 14.54 | 0.95 |
|  | Average | 21.75 | 21.86 | 21.80 |  |  |
| CFF | 0-10 | 11.55 | 12.00 | 11.10 | 11.55 | 0.45 |
|  | 10-20 | 11.00 | 7.91 | 10.39 | 9.77 | 1.64 |
|  | 20-30 | 6.70 | 9.43 | 8.06 | 8.06 | 1.36 |
|  | Average | 9.75 | 9.78 | 9.85 |  |  |
| MCBF | 0-10 | 8.87 | 9.22 | 12.05 | 10.05 | 1.74 |
|  | 10-20 | 3.49 | 3.36 | 2.37 | 3.08 | 0.61 |
|  | 20-30 | 1.69 | 1.80 | 1.49 | 1.66 | 0.15 |
|  | Average | 4.68 | 4.79 | 5.31 |  |  |
| EBF | 0-10 | 19.83 | 17.74 | 17.52 | 18.36 | 1.27 |
|  | 10-20 | 11.95 | 10.00 | 8.37 | 10.11 | 1.79 |
|  | 20-30 | 8.00 | 7.99 | 5.46 | 7.15 | 1.47 |
|  | Average | 13.26 | 11.91 | 10.45 |  |  |
|  |  |  |  |  |  |  |
| DOC(mg/kg) |  |  |  |  |  |  |
|  | Soil depth (cm) | Repeat1 | Repeat2 | Repeat3 | Average | STDE |
| SH | 0-10 | 91.70 | 92.85 | 92.28 | 92.28 | 0.57 |
|  | 10-20 | 57.70 | 65.45 | 61.58 | 61.58 | 3.88 |
|  | 20-30 | 48.47 | 51.55 | 50.01 | 50.01 | 1.54 |
|  | Average | 65.96 | 69.95 | 67.95 | 67.95 |  |
| BF | 0-10 | 128.65 | 136.4 | 132.53 | 132.53 | 3.88 |
|  | 10-20 | 76.65 | 70.15 | 73.40 | 73.40 | 3.25 |
|  | 20-30 | 58.6 | 73.15 | 65.88 | 65.88 | 7.28 |
|  | Average | 87.97 | 93.23 | 90.6 | 90.60 |  |
| CFF | 0-10 | 77.13 | 70.95 | 83.3 | 77.13 | 6.18 |
|  | 10-20 | 60.9 | 57.35 | 59.13 | 59.13 | 1.78 |
|  | 20-30 | 46.08 | 57.35 | 51.72 | 51.72 | 5.64 |
|  | Average | 61.37 | 61.88 | 64.72 | 62.66 |  |
| MCBF | 0-10 | 97.4 | 96.05 | 96.73 | 96.73 | 0.68 |
|  | 10-20 | 53.45 | 35.075 | 47.855 | 45.46 | 9.42 |
|  | 20-30 | 27.37 | 33.01 | 21.73 | 27.37 | 5.64 |
|  | Average | 59.41 | 54.71 | 55.44 | 56.52 |  |
| EBF | 0-10 | 118.63 | 128.55 | 108.7 | 118.63 | 9.93 |
|  | 10-20 | 78.15 | 74.45 | 60.85 | 71.15 | 9.11 |
|  | 20-30 | 73.05 | 56.8 | 64.93 | 64.93 | 8.13 |
|  | Average | 89.94 | 86.6 | 78.16 | 84.90 |  |

| MBC(mg/kg) | Soil depth (cm) | Repeat1 | Repeat2 | Repeat3 | Average | STDE |
| --- | --- | --- | --- | --- | --- | --- |
| SH | 0-10 | 184.92 | 161.33 | 173.13 | 173.13 | 11.79 |
|  | 10-20 | 124.08 | 142.84 | 133.46 | 133.46 | 9.38 |
|  | 20-30 | 93.97 | 91.03 | 92.50 | 92.50 | 1.47 |
|  |  | 134.32 | 131.73 | 133.03 |  |  |
| BF | 0-10 | 376.48 | 372.53 | 374.50 | 374.50 | 1.97 |
|  | 10-20 | 139.27 | 156.37 | 147.82 | 147.82 | 8.55 |
|  | 20-30 | 153.15 | 171.24 | 162.20 | 162.20 | 9.05 |
|  |  | 222.96 | 233.38 | 228.17 |  |  |
| CFF | 0-10 | 119.31 | 105.43 | 102.64 | 109.13 | 8.93 |
|  | 10-20 | 145.01 | 99.00 | 159.22 | 134.41 | 31.48 |
|  | 20-30 | 127.12 | 104.43 | 149.81 | 127.12 | 22.69 |
|  |  | 130.48 | 102.95 | 137.22 |  |  |
| MCBF | 0-10 | 111.21 | 132.38 | 123.15 | 122.25 | 10.61 |
|  | 10-20 | 96.63 | 87.21 | 77.78 | 87.21 | 9.43 |
|  | 20-30 | 57.65 | 50.89 | 49.38 | 52.64 | 4.41 |
|  |  | 88.50 | 90.16 | 83.44 |  |  |
| EBF | 0-10 | 232.40 | 268.74 | 196.05 | 232.40 | 36.34 |
|  | 10-20 | 170.54 | 153.42 | 147.08 | 157.01 | 12.13 |
|  | 20-30 | 137.34 | 121.61 | 115.62 | 124.85 | 11.22 |
| EOC(g/kg) | Soil depth (cm) | Repeat1 | Repeat2 | Repeat3 | Average | STDE |
| SH | 0-10 | 4.22 | 3.39 | 3.80 | 3.80 | 0.41 |
|  | 10-20 | 0.97 | 1.33 | 1.15 | 1.15 | 0.18 |
|  | 20-30 | 0.84 | 0.88 | 0.86 | 0.86 | 0.02 |
|  |  | 2.01 | 1.87 | 1.94 |  |  |
| BF | 0-10 | 4.34 | 4.66 | 4.50 | 4.50 | 0.16 |
|  | 10-20 | 2.84 | 2.47 | 2.65 | 2.65 | 0.19 |
|  | 20-30 | 2.13 | 2.78 | 2.45 | 2.45 | 0.33 |
|  |  | 3.10 | 3.30 | 3.20 |  |  |
| CFF | 0-10 | 2.59 | 2.52 | 2.50 | 2.54 | 0.05 |
|  | 10-20 | 2.49 | 2.24 | 2.31 | 2.35 | 0.13 |
|  | 20-30 | 1.29 | 1.93 | 1.57 | 1.60 | 0.32 |
|  |  | 2.13 | 2.23 | 2.13 |  |  |
| MCBF | 0-10 | 2.22 | 1.93 | 2.56 | 2.24 | 0.31 |
|  | 10-20 | 0.71 | 0.78 | 0.86 | 0.78 | 0.08 |
|  | 20-30 | 0.34 | 0.25 | 0.24 | 0.28 | 0.05 |
|  |  | 1.09 | 0.99 | 1.22 |  |  |
| EBF | 0-10 | 4.78 | 3.76 | 3.64 | 4.06 | 0.62 |
|  | 10-20 | 2.33 | 2.29 | 1.71 | 2.11 | 0.35 |
|  | 20-30 | 1.45 | 1.38 | 1.42 | 1.42 | 0.04 |
|  |  | 2.86 | 2.48 | 2.26 |  |  |

**S3 Table. The proportion of LOCFs (DOC, MBC, and EOC) in total SOC (%) of the five vegetation types.**

| DOC/SOC(%) | Soil depth (cm) | Repeat1 | Repeat2 | Repeat3 | Average | STDE |
| --- | --- | --- | --- | --- | --- | --- |
| SH | 0-10 | 0.52 | 0.72 | 0.61 | 0.61 | 0.10 |
|  | 10-20 | 1.03 | 1.04 | 1.04 | 1.04 | 0.01 |
|  | 20-30 | 1.01 | 1.20 | 1.10 | 1.10 | 0.09 |
|  |  | 0.85 | 0.99 | 0.91 | 0.92 | 0.07 |
| BF | 0-10 | 0.40 | 0.39 | 0.40 | 0.40 | 0.01 |
|  | 10-20 | 0.39 | 0.47 | 0.42 | 0.43 | 0.04 |
|  | 20-30 | 0.43 | 0.35 | 0.38 | 0.39 | 0.04 |
|  |  | 0.41 | 0.40 | 0.40 | 0.40 | 0.03 |
| CFF | 0-10 | 0.67 | 0.59 | 0.75 | 0.67 | 0.08 |
|  | 10-20 | 0.55 | 0.73 | 0.57 | 0.62 | 0.09 |
|  | 20-30 | 0.69 | 0.61 | 0.64 | 0.65 | 0.04 |
|  |  | 0.64 | 0.64 | 0.65 | 0.64 | 0.07 |
| MCBF | 0-10 | 1.10 | 1.04 | 0.80 | 0.98 | 0.16 |
|  | 10-20 | 1.53 | 1.04 | 2.02 | 1.53 | 0.49 |
|  | 20-30 | 1.62 | 1.84 | 1.46 | 1.64 | 0.19 |
|  |  | 1.42 | 1.31 | 1.42 | 1.38 | 0.28 |
| EBF | 0-10 | 0.60 | 0.72 | 0.62 | 0.65 | 0.07 |
|  | 10-20 | 0.65 | 0.74 | 0.73 | 0.71 | 0.05 |
|  | 20-30 | 0.91 | 0.71 | 1.19 | 0.94 | 0.14 |
|  |  | 0.72 | 0.73 | 0.85 | 0.76 | 0.09 |
|  |  |  |  |  |  |  |
| MBC/SOC(%) | Soil depth (cm) | Repeat1 | Repeat2 | Repeat3 | Average | STDE |
| SH | 0-10 | 1.06 | 1.24 | 1.14 | 1.14 | 0.09 |
|  | 10-20 | 2.21 | 2.27 | 2.25 | 2.25 | 0.03 |
|  | 20-30 | 1.96 | 2.12 | 2.03 | 2.04 | 0.08 |
|  |  | 1.74 | 1.88 | 1.80 | 1.81 | 0.07 |
| BF | 0-10 | 1.18 | 1.06 | 1.12 | 1.12 | 0.06 |
|  | 10-20 | 0.70 | 1.04 | 0.85 | 0.86 | 0.17 |
|  | 20-30 | 1.13 | 0.81 | 0.93 | 0.96 | 0.16 |
|  |  | 1.00 | 0.97 | 0.97 | 0.98 | 0.13 |
| CFF | 0-10 | 1.03 | 0.88 | 0.92 | 0.95 | 0.08 |
|  | 10-20 | 1.32 | 1.25 | 1.53 | 1.37 | 0.15 |
|  | 20-30 | 1.90 | 1.11 | 1.86 | 1.62 | 0.45 |
|  |  | 1.42 | 1.08 | 1.44 | 1.31 | 0.22 |
| MCBF | 0-10 | 1.25 | 1.44 | 1.02 | 1.24 | 0.21 |
|  | 10-20 | 2.77 | 2.60 | 3.28 | 2.88 | 0.35 |
|  | 20-30 | 3.42 | 2.83 | 3.31 | 3.19 | 0.31 |
|  |  | 2.48 | 2.29 | 2.53 | 2.43 | 0.29 |
| EBF | 0-10 | 1.17 | 1.51 | 1.12 | 1.27 | 0.21 |
|  | 10-20 | 1.43 | 1.53 | 1.76 | 1.57 | 0.17 |
|  | 20-30 | 1.72 | 1.52 | 2.12 | 1.79 | 0.30 |
|  |  | 1.44 | 1.52 | 1.66 | 1.54 | 0.23 |
|  |  |  |  |  |  |  |
| EOC/SOC(%) | Soil depth (cm) | Repeat1 | Repeat2 | Repeat3 | Average | STDE |
| SH | 0-10 | 24.08 | 26.12 | 24.95 | 25.05 | 1.02 |
|  | 10-20 | 17.30 | 21.16 | 19.34 | 19.26 | 1.93 |
|  | 20-30 | 17.55 | 20.49 | 18.94 | 18.99 | 1.47 |
|  |  | 19.64 | 22.59 | 21.08 | 21.10 | 1.47 |
| BF | 0-10 | 13.63 | 13.28 | 13.44 | 13.45 | 0.18 |
|  | 10-20 | 14.33 | 16.44 | 15.24 | 15.33 | 1.06 |
|  | 20-30 | 15.63 | 13.11 | 14.10 | 14.28 | 1.27 |
|  |  | 14.53 | 14.27 | 14.26 | 14.35 | 0.84 |
| CFF | 0-10 | 22.44 | 21.02 | 22.53 | 22.00 | 0.84 |
|  | 10-20 | 22.63 | 28.27 | 22.25 | 24.38 | 3.37 |
|  | 20-30 | 19.32 | 20.51 | 19.42 | 19.75 | 0.66 |
|  |  | 21.46 | 23.27 | 21.40 | 22.04 | 1.63 |
| MCBF | 0-10 | 25.03 | 20.92 | 21.23 | 22.39 | 2.29 |
|  | 10-20 | 20.20 | 23.22 | 36.11 | 26.51 | 8.45 |
|  | 20-30 | 20.17 | 13.67 | 16.35 | 16.73 | 3.27 |
|  |  | 21.80 | 19.27 | 24.56 | 21.88 | 4.67 |
| EBF | 0-10 | 24.10 | 21.22 | 20.78 | 22.03 | 1.80 |
|  | 10-20 | 19.52 | 22.92 | 20.47 | 20.97 | 1.76 |
|  | 20-30 | 18.18 | 17.30 | 26.02 | 20.50 | 4.80 |
|  |  | 20.60 | 20.48 | 22.42 | 21.17 | 2.79 |

**S4 Table. LOCFs (DOC, MBC, and EOC) stocks in the different vegetation types.**

| DOC stock(t ha^-1^) | Soil depth (cm) | Repeat1 | Repeat2 | Repeat3 | Average | STDE |
| --- | --- | --- | --- | --- | --- | --- |
| SH | 0-10 | 0.15 | 0.17 | 0.16 | 0.16 | 0.01 |
|  | 10-20 | 0.10 | 0.13 | 0.11 | 0.11 | 0.02 |
|  | 20-30 | 0.10 | 0.10 | 0.10 | 0.10 | 0.00 |
|  |  | 0.35 | 0.40 | 0.37 | 0.37 | 0.03 |
| BF | 0-10 | 0.17 | 0.17 | 0.17 | 0.17 | 0.00 |
|  | 10-20 | 0.109 | 0.111 | 0.110 | 0.110 | 0.00 |
|  | 20-30 | 0.108 | 0.105 | 0.108 | 0.107 | 0.00 |
|  |  | 0.39 | 0.39 | 0.39 | 0.39 |  |
| CFF | 0-10 | 0.12 | 0.10 | 0.15 | 0.12 | 0.02 |
|  | 10-20 | 0.09 | 0.10 | 0.09 | 0.09 | 0.01 |
|  | 20-30 | 0.08 | 0.07 | 0.09 | 0.08 | 0.01 |
|  |  | 0.29 | 0.28 | 0.32 | 0.30 |  |
| MCBF | 0-10 | 0.21 | 0.19 | 0.15 | 0.18 | 0.03 |
|  | 10-20 | 0.11 | 0.07 | 0.07 | 0.09 | 0.02 |
|  | 20-30 | 0.05 | 0.06 | 0.04 | 0.05 | 0.01 |
|  |  | 0.37 | 0.32 | 0.27 | 0.32 | 0.06 |
| EBF | 0-10 | 0.17 | 0.19 | 0.18 | 0.18 | 0.01 |
|  | 10-20 | 0.13 | 0.14 | 0.12 | 0.13 | 0.01 |
|  | 20-30 | 0.15 | 0.11 | 0.14 | 0.13 | 0.02 |
|  |  | 0.44 | 0.44 | 0.44 | 0.44 |  |
|  |  |  |  |  |  |  |
| MBC stock(t ha^-1^) | Soil depth (cm) | Repeat1 | Repeat2 | Repeat3 | Average | STDE |
| SH | 0-10 | 0.31 | 0.29 | 0.30 | 0.30 | 0.01 |
|  | 10-20 | 0.21 | 0.29 | 0.25 | 0.25 | 0.04 |
|  | 20-30 | 0.19 | 0.17 | 0.18 | 0.18 | 0.01 |
|  |  | 0.71 | 0.75 | 0.73 | 0.73 | 0.06 |
| BF | 0-10 | 0.50 | 0.47 | 0.48 | 0.48 | 0.02 |
|  | 10-20 | 0.20 | 0.25 | 0.22 | 0.22 | 0.02 |
|  | 20-30 | 0.28 | 0.24 | 0.27 | 0.26 | 0.02 |
|  |  | 0.98 | 0.96 | 0.97 |  |  |
| CFF | 0-10 | 0.19 | 0.15 | 0.18 | 0.175 | 0.02 |
|  | 10-20 | 0.20 | 0.17 | 0.25 | 0.205 | 0.04 |
|  | 20-30 | 0.23 | 0.19 | 0.18 | 0.201 | 0.03 |
|  |  | 0.63 | 0.51 | 0.60 |  |  |
| MCBF | 0-10 | 0.24 | 0.26 | 0.19 | 0.23 | 0.03 |
|  | 10-20 | 0.20 | 0.18 | 0.12 | 0.17 | 0.04 |
|  | 20-30 | 0.11 | 0.09 | 0.10 | 0.10 | 0.01 |
|  |  | 0.55 | 0.53 | 0.41 |  |  |
| EBF | 0-10 | 0.33 | 0.39 | 0.33 | 0.35 | 0.03 |
|  | 10-20 | 0.27 | 0.29 | 0.28 | 0.28 | 0.01 |
|  | 20-30 | 0.28 | 0.24 | 0.25 | 0.25 | 0.02 |
|  |  | 0.88 | 0.91 | 0.86 |  |  |
|  |  |  |  |  |  |  |
| EOC stock(t ha^-1^) | Soil depth (cm) | Repeat1 | Repeat2 | Repeat3 | Average | STDE |
| SH | 0-10 | 7.09 | 6.17 | 6.66 | 6.64 | 0.46 |
|  | 10-20 | 1.63 | 2.71 | 2.14 | 2.16 | 0.54 |
|  | 20-30 | 1.73 | 1.63 | 1.68 | 1.68 | 0.05 |
|  |  | 10.44 | 10.51 | 10.47 | 10.48 | 1.05 |
| BF | 0-10 | 5.77 | 5.82 | 5.80 | 5.80 | 0.03 |
|  | 10-20 | 4.03 | 3.89 | 3.98 | 3.97 | 0.07 |
|  | 20-30 | 3.91 | 3.97 | 4.01 | 3.96 | 0.05 |
|  |  | 13.71 | 13.69 | 13.79 | 13.73 |  |
| CFF | 0-10 | 4.12 | 3.71 | 4.38 | 4.07 | 0.34 |
|  | 10-20 | 3.51 | 3.76 | 3.56 | 3.61 | 0.13 |
|  | 20-30 | 2.38 | 2.51 | 2.63 | 2.51 | 0.12 |
|  |  | 10.01 | 9.98 | 10.57 | 10.19 |  |
| MCBF | 0-10 | 3.82 | 3.78 | 3.99 | 3.86 | 0.11 |
|  | 10-20 | 1.46 | 1.61 | 1.34 | 1.47 | 0.14 |
|  | 20-30 | 0.65 | 0.44 | 0.47 | 0.52 | 0.11 |
|  |  | 5.93 | 5.84 | 5.80 | 5.86 |  |
| EBF | 0-10 | 6.78 | 5.42 | 6.08 | 6.10 | 0.68 |
|  | 10-20 | 3.73 | 4.31 | 3.29 | 3.78 | 0.51 |
|  | 20-30 | 2.92 | 2.71 | 3.04 | 2.89 | 0.17 |
|  |  | 13.44 | 12.44 | 12.41 | 12.76 |  |

**S5 Table. SOC pool indexes (L, LI, CPI, and CPMI) of the different vegetation types.**

| CPI |  |  |  |  |  |  |
| --- | --- | --- | --- | --- | --- | --- |
|  | Soil depth (cm) | Repeat1 | Repeat2 | Repeat3 | Average | STDE |
| BF | 0-10 | 1.82 | 2.70 | 2.19 | 2.24 | 0.45 |
|  | 10-20 | 3.54 | 2.39 | 2.93 | 2.95 | 0.57 |
|  | 20-30 | 2.83 | 4.92 | 3.82 | 3.86 | 0.85 |
|  |  | 2.73 | 3.34 | 2.98 | 3.02 | 0.62 |
| CFF | 0-10 | 0.66 | 0.92 | 0.73 | 0.77 | 0.14 |
|  | 10-20 | 1.96 | 1.26 | 1.75 | 1.66 | 0.36 |
|  | 20-30 | 1.40 | 2.19 | 1.77 | 1.79 | 0.40 |
|  |  | 1.34 | 1.46 | 1.42 | 1.40 | 0.30 |
| MCBF | 0-10 | 0.51 | 0.71 | 0.79 | 0.67 | 0.15 |
|  | 10-20 | 0.62 | 0.53 | 0.40 | 0.52 | 0.11 |
|  | 20-30 | 0.35 | 0.42 | 0.33 | 0.37 | 0.05 |
|  |  | 0.49 | 0.55 | 0.51 | 0.52 | 0.10 |
| EBF | 0-10 | 1.13 | 1.37 | 1.15 | 1.22 | 0.13 |
|  | 10-20 | 2.13 | 1.59 | 1.41 | 1.71 | 0.38 |
|  | 20-30 | 1.67 | 1.86 | 1.20 | 1.57 | 0.34 |
|  |  | 1.64 | 1.61 | 1.25 | 1.50 |  |
| LI | Soil depth (cm) | Repeat1 | Repeat2 | Repeat3 | Average | STDE |
| BF | 0-10 | 0.50 | 0.43 | 0.47 | 0.47 | 0.03 |
|  | 10-20 | 0.80 | 0.73 | 0.75 | 0.76 | 0.03 |
|  | 20-30 | 0.87 | 0.59 | 0.70 | 0.72 | 0.14 |
|  |  | 0.69 | 0.57 | 0.62 | 0.65 | 0.07 |
| CFF | 0-10 | 0.91 | 0.75 | 0.87 | 0.85 | 0.08 |
|  | 10-20 | 1.40 | 1.47 | 1.19 | 1.35 | 0.14 |
|  | 20-30 | 1.12 | 1.00 | 1.03 | 1.05 | 0.06 |
|  |  | 1.11 | 1.04 | 1.02 | 1.08 | 0.10 |
| MCBF | 0-10 | 1.05 | 0.75 | 0.81 | 0.87 | 0.16 |
|  | 10-20 | 1.21 | 1.13 | 1.17 | 1.17 | 0.04 |
|  | 20-30 | 1.19 | 0.61 | 0.84 | 0.88 | 0.19 |
|  |  | 1.14 | 0.82 | 0.92 | 0.97 | 0.13 |
| EBF | 0-10 | 1.00 | 0.76 | 0.79 | 0.85 | 0.13 |
|  | 10-20 | 1.16 | 1.11 | 1.07 | 1.11 | 0.04 |
|  | 20-30 | 1.04 | 0.81 | 1.51 | 1.12 | 0.35 |
|  |  | 1.07 | 0.89 | 1.12 | 1.03 | 0.18 |
| CPMI |  |  |  |  |  |  |
|  | Soil depth (cm) | Repeat1 | Repeat2 | Repeat3 | Average | STDE |
| BF | 0-10 | 90.43 | 117.08 | 102.56 | 103.36 | 13.34 |
|  | 10-20 | 282.76 | 175.08 | 219.67 | 225.84 | 54.10 |
|  | 20-30 | 246.41 | 288.25 | 268.24 | 267.63 | 20.92 |
|  |  | 206.53 | 193.47 | 196.82 | 198.94 | 29.46 |
| CFF | 0-10 | 60.16 | 69.62 | 63.69 | 64.49 | 4.78 |
|  | 10-20 | 274.51 | 184.97 | 208.79 | 222.76 | 46.38 |
|  | 20-30 | 156.96 | 219.49 | 182.65 | 186.37 | 31.43 |
|  |  | 163.88 | 158.03 | 151.71 | 157.87 | 27.53 |
| MCBF | 0-10 | 53.31 | 53.18 | 64.10 | 56.86 | 6.27 |
|  | 10-20 | 75.47 | 60.29 | 46.68 | 60.81 | 4.40 |
|  | 20-30 | 41.66 | 25.69 | 27.44 | 31.60 | 8.76 |
|  |  | 56.81 | 46.39 | 46.08 | 49.76 | 6.48 |
| EBF | 0-10 | 113.31 | 104.15 | 90.66 | 102.70 | 11.40 |
|  | 10-20 | 247.38 | 176.50 | 151.31 | 191.73 | 49.81 |
|  | 20-30 | 173.92 | 150.88 | 180.51 | 168.43 | 15.56 |
|  |  | 178.20 | 143.84 | 140.82 | 154.29 | 25.59 |
| L | Soil depth (cm) | Repeat1 | Repeat2 | Repeat3 | Average | STDE |
| BF | 0-10 | 0.16 | 0.15 | 0.16 | 0.16 | 0.00 |
|  | 10-20 | 0.17 | 0.20 | 0.18 | 0.18 | 0.01 |
|  | 20-30 | 0.19 | 0.15 | 0.16 | 0.17 | 0.02 |
|  |  | 0.17 | 0.17 | 0.17 | 0.17 | 0.01 |
| CFF | 0-10 | 0.29 | 0.27 | 0.29 | 0.28 | 0.01 |
|  | 10-20 | 0.29 | 0.39 | 0.29 | 0.32 | 0.06 |
|  | 20-30 | 0.24 | 0.26 | 0.24 | 0.25 | 0.01 |
|  |  | 0.27 | 0.31 | 0.27 | 0.28 | 0.03 |
| MCBF | 0-10 | 0.33 | 0.26 | 0.27 | 0.29 | 0.04 |
|  | 10-20 | 0.25 | 0.30 | 0.28 | 0.28 | 0.02 |
|  | 20-30 | 0.25 | 0.16 | 0.20 | 0.20 | 0.05 |
|  |  | 0.28 | 0.24 | 0.25 | 0.26 | 0.04 |
| EBF | 0-10 | 0.32 | 0.27 | 0.26 | 0.28 | 0.03 |
|  | 10-20 | 0.24 | 0.30 | 0.26 | 0.27 | 0.03 |
|  | 20-30 | 0.22 | 0.21 | 0.35 | 0.26 | 0.08 |
|  |  | 0.26 | 0.26 | 0.29 | 0.27 | 0.05 |
