# Supplementary figures and images for: Responses of soil labile organic carbon stocks and the carbon pool management index to different vegetation restoration types in the Danxia landform region of southwest China
Source: PLoS One. 2025 Feb 25;20(2):e0318195. doi: 10.1371/journal.pone.0318195 (PMC11856273; doi:10.1371/journal.pone.0318195)

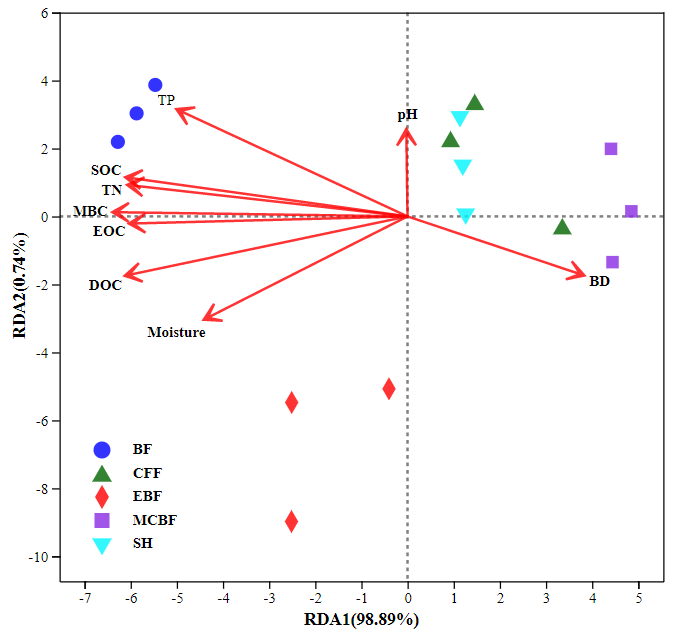

Supplement: S1 Text — S1 Table. Mean (±SE, n = 3) soil basic physical and chemical properties in the five vegetation types. S2 Table.Two-way ANOVA of the effects of different vegetation types (VT) and soil depths (SD) on SOC and LOCFs. S3 Table.The proportion of LOCFs (DOC, MBC, and EOC) in total SOC (%) of the five vegetation types. S4 Table. LOCFs (DOC, MBC, and EOC) stocks in the different vegetation types. S5 Table. SOC pool indexes (L, LI, CPI, and CPMI) of the different vegetation types. S1 Fig. SOC concentration (a) and stocks (b) in each soil layer of the different vegetation types. S2 Fig. LOCF concentrations of the different vegetation types. a) DOC, b) MBC, and c) EOC. S3 Fig. Redundancy analysis of soil carbon fractions and physicochemical properties. (ZIP) [file pone.0318195.s001.zip › Supporting information/Figures/Fig 3.tif]
