# Supplementary material for: Responses of soil labile organic carbon stocks and the carbon pool management index to different vegetation restoration types in the Danxia landform region of southwest China
Source: PLoS One. 2025 Feb 25;20(2):e0318195. doi: 10.1371/journal.pone.0318195 (PMC11856273; doi:10.1371/journal.pone.0318195)
Supplement: Attachment — Submitted filename: Response to Reviewers and editors.docx [file pone.0318195.s002.docx]

Dear Reviewers and Editors :

Thank you very much for your constructive comments and valuable recommendations concerning our manuscript entitled “Responses of soil labile organic carbon stocks and the carbon pool management index to different vegetation restoration types in the Danxia landform region of southwest China” (manuscript number: PONE-D-24-34559). These comments are very helpful for revising and improving our paper, as well as the important guiding significance to our researches. We have carefully revised the manuscript according to your suggestions which we hope meet with approval. Revised portion are marked in red in the revised manuscript (Revised manuscript with Track changes). Our response to the comments are listed below:

**Response to reviewer 1:**

**Comment 1:** Abstract
- L15: Delete ‘in China’s Danxia region’
- L34: exhibit?

**Reply:**Thank you very much for the advice. We have deleted ‘in China’s Danxia region’ in line 15 and correct the incorrect word "exbibit" to"exhibited" in line 34 in our revised manuscript, please see our revised manuscript page1, lines 15; page2, 33.

**Comment 2:** Introduction
- L77: Blair and Lefroy [20]

**Reply:**Thank you very much. We have amended "Blair and Lefroy" to" Blair et al.", please see the introduction in our revised manuscript page4, lines 74.

**Comment 3:** Materials and methods
- L153: by Ghani et al. [29].
**Reply:**Thank you very much. We have added “Ghani et al.” in L157, please see the ‘Materials and methods’ section in our revised manuscript page 8, lines 157.

**Comment 4:** Result
- L259: Check the use of abbreviations. Does lability (L) indicate ‘Lability of C’ shown in L176’?

**Reply:** We scrutinized the use of abbreviations. Yes, the lability (L) in L259 indicate ‘Lability of C’ shown in L176. To make it clearer to readers, we have added the abbreviation ‘(L)’ to formula (3) and changed ‘lability (L)’ in L259 to ‘lability of C (L)’. The following is the revised formula(3) :

$$Lability\text{ }of\text{ }C\text{ }(L)=\frac{EOC\text{ }(g\cdot kg^{-1})}{SOC(g\cdot kg^{-1})-EOC\text{ }(g\cdot kg^{-1})}$$

Please see the ‘Result’ section in our revised manuscript page 10, lines 179 and page 15, lines 281.

**Comment 5:** Discussion
- L309: Table 2
- L361: Filep et al. [66]
- L375: by Pang et al. [24]; Chen et al. [70]
**Reply:**Thank you very much. We have corrected ‘Table 1’to ‘Table 2’ in L309. Filep et al.(2015) [66] in L361 and by [24], Chen et al. (2019)[70] in L375 also were modified into ‘Filep et al. [66]’ in L361 and ‘by Pang et al. [24]; Chen et al. [70]’ in L375. Please see the ‘Discussion’ section in our revised manuscript page 18,line 340; page 21, line 405 and page 21, line 411.

**Comment 6:** Tables & Figures
- Table and figure legends should be improved, including statistic description.

**Reply:** We have modified the tables and figures to make it clear which letters are capitalized and which are lowercase, and to specify clearly that the error measurement is standard error (SE). The statistical description is clearer. In addition, each abbreviation is fully defined in each table and figure legends. Please see the tables and figures in our revised manuscript.

**Comment 7:**Reference list
- It should be revised. Please check the submission guidelines of the PLOS ONE journal.

**Reply:**We have revised the references format according to the submission guidelines of the PLOS ONE journal, please see the revised Reference section, page 27-32, line 533-767.

**Response to reviewer #2:**

**Comment 1**:The introduction could be improved by a more detailed discussion on the gaps in current research specifically relating to SOC dynamics in the Danxia landform under different vegetation restorations. References to previous studies are appropriately included, yet a clearer linkage between these studies and the current research question would enhance the manuscript's introduction.

**Reply:** We added the sentence ‘As for the Danxia landform area, people’s research mainly focuses on the vegetation, biodiversity, geological structure and tourism development [26,27,3]’ in our revised manuscript, page 5, line 99-101, to illustrate the previous research on Danxia and highlight the signifiance of our research.

The refenrences were added:

[26] Yang J, Xiong K, Xiao S. Spatial temporal variation of vegetation coverage of reddish landform in Chishui Cityi.Yangtze River, 2018, 49 (18): 38-44. <https://doi.org/10.16232/j.cnki.1001-4179.2018.18.009.>
[27] Meng, S. Y., Chen, B., & Sodmergen. (2023). Sedum danxiacola (Crassulaceae), an endemic new species from the Danxia landform, China. Kew Bulletin, 78(4), 673-681. https://doi.org/10.1007/S12225-023-10122-Y.

**Comment 2:**The discussion would benefit from a more detailed comparison with other similar ecosystems globally, which would provide a broader context for the findings.
**Reply:**In the discussion section, we added the following:

However, except for BF, SOC concentrations in the other four vegetation types in surface soil layer (0-10 cm) were 10.05-18.36 g·kg^-1^, lower than those of the Danxia natural forest in southeast China (24.32 g·kg^-1^) [58]. Moreover, total SOC stocks in EBF, CFF, SH, and MCBF were 26.18-60.13 t ha^−1^, lower than that in natural regeneration of secondary forest (74.07 t ha^−1^) in Karst region of southwest China at a depth of 0-30 cm studied by Pang et al. (2019)[23].

Please see the revised manuscript, page 20, line 373-378.

**Comment 3:** Statistical analysis: Did you check residual normality? Please explain in the manuscript.

**Reply:** We have checked residual normality of data in the statistical analysis, which we have explained in our revised manuscript, please see page 10,183-184.

**Comment 4:** Figures and Tables: Clarify which letters are capitalized and which are lowercase, and specify whether the error measurement is standard deviation (SD) or standard error (SE). Please provide the full forms of all abbreviations used. Each figure and table should be self-explanatory, ensuring that the reader can understand the content without referring to the main text.

**Reply:** We have modified the tables and figures to make it clear which letters are capitalized and which are lowercase, and to specify clearly that the error measurement is standard error (SE). The statistical description is clearer. In addition, each abbreviation is fully defined in each table and figure legends. Please see the tables and figures in our revised manuscript.

**Comment 5:** Redundancy analysis: Please specify which software was used to perform the RDA in your methods section. Providing the exact software and version used will help readers replicate your results. Additionally, make sure to mention any specific settings, packages, or libraries applied during the analysis. The abbreviations such as "SOC", "TN", "MBC", "EOC", "DOC", "BD", "TP", and "pH" are not explained within the figure. For clarity, each abbreviation should be fully defined in either the caption or the legend. "RDA1 (98.89%)" and "RDA2 (0.74%)" show the percentage of the variance explained by the respective axes, which is good, but a clearer explanation of what these represent in the context of the study might be needed in the caption.

**Reply:** We have clarified in the statistical analysis section that the Redundancy Analysis (RDA) is carried out on the online tool of Majorbio Cloud Platform (https://cloud. Majorbio.com/page/tools/), please see the revised manuscript page 10, line 190-191. The all abbreviations in the figure (RDA) also are explained in figure legend, please see the revised manuscript page 17, line 311-314. For "RDA1 (98.89%)" and "RDA2 (0.74%)" showing the percentage of variance interpreted by the respective axes, we added an explanation“As observed, the two axes effectively illustrate the correlation between soil C fractions and its physicochemical properties, with the first axis serving as a primary determinant”, see the revised version, please see the the revised manuscript page 16, line 300-301.

**Response to the editor’s comments:**

1. Please ensure that your manuscript meets PLOS ONE's style requirements, including those for file naming. The PLOS ONE style templates can be found at：https://journals.plos.org/plosone/s/file?id=wjVg/PLOSOne_formatting_sample_main_body.pdf and

https://journals.plos.org/plosone/s/file?id=ba62/PLOSOne_formatting_sample_title_authors_affiliations.pdf

**Reply:** We have modified the format according to the PLOS ONE style. Please see the revised manuscript.

2. We noticed you have some minor occurrence of overlapping text with the following previous publication(s), which needs to be addressed:

Responses of soil labile organic carbon fractions and stocks to different vegetation restoration strategies in degraded karst ecosystems of southwest China - https://doi.org/10.1016/j.ecoleng.2019.08.008

Build-up of labile, non-labile carbon fractions under fourteen-year-old bamboo plantations in the Himalayan foothills -https://doi.org/10.1016/j.heliyon.2021.e07850

(Among others)

In your revision ensure you cite all your sources (including your own works), and quote or rephrase any duplicated text outside the methods section. Further consideration is dependent on these concerns being addressed.

**Reply:** We have revised and restated the duplicates you mentioned, and checked all cited sources (including our own works). Please see the revised manuscript.

3. In your Methods section, please provide additional information regarding the permits you obtained for the work. Please ensure you have included the full name of the authority that approved the field site access and, if no permits were required, a brief statement explaining why.

**Reply:** We added “Our research obtained the forestry administrative license of Xishui National Nature Reserve Administration of Guizhou Province.”Please see the “Sample collection and chemical analyses” in our Methods section, page 7, line 128-129.

We have included the full name of the authority that approved the field site access.

4. Please state what role the funders took in the study. If the funders had no role, please state: "The funders had no role in study design, data collection and analysis, decision to publish, or preparation of the manuscript. If this statement is not correct you must amend it as needed.

**Reply:** Our funding is funded by government agencies. Funding agencies provide financial support for our research design, data collection and analysis, publication decisions or manuscript preparation.

5. We note that you have provided funding information that is not currently declared in your Funding Statement. However, funding information should not appear in the Acknowledgments section or other areas of your manuscript. We will only publish funding information present in the Funding Statement section of the online submission form. Please remove any funding-related text from the manuscript and let us know how you would like to update your Funding Statement. Currently, your Funding Statement reads as follows: "Guizhou Provincial Basic Research Program (Natural Science) (Qiankehejichu-ZK[2024]yiban 674 and 691) and Scientific Research Project of Zunyi Normal University (Zunshi BS [2019]30)."

**Reply:** We have already deleted the funding information in the Acknowledgments section. **We have provided a funding statement in cover letter; we wish editor change the online submission form on our behalf,** our Funding Statement as follows: "Guizhou Provincial Basic Research Program (Natural Science) (Qiankehejichu-ZK[2024]yiban 674 and 691) and Scientific Research Project of Zunyi Normal University (Zunshi BS [2019]30)." **.**

6. Please confirm at this time whether or not your submission contains all raw data required to replicate the results of your study. Authors must share the “minimal data set” for their submission. PLOS defines the minimal data set to consist of the data required to replicate all study findings reported in the article, as well as related metadata and methods.

# **Reply:** We confirm at this time our submission contains all raw data required to replicate the results of our study. The raw data please see the “Supporting information”.

7. If there are ethical or legal restrictions on sharing a de-identified data set, please explain them in detail (e.g., data contain potentially sensitive information, data are owned by a third-party organization, etc.) and who has imposed them (e.g., an ethics committee). Please also provide contact information for a data access committee, ethics committee, or other institutional body to which data requests may be sent. If data are owned by a third party, please indicate how others may request data access.

**Reply:** There are no ethical or legal restrictions on the data we submit.

8. PLOS requires an ORCID iD for the corresponding author in Editorial Manager on papers submitted after December 6th, 2016. Please ensure that you have an ORCID iD and that it is validated in Editorial Manager. To do this, go to ‘Update my Information’ (in the upper left-hand corner of the main menu), and click on the Fetch/Validate link next to the ORCID field. This will take you to the ORCID site and allow you to create a new iD or authenticate a pre-existing iD in Editorial Manager.

**Reply:** The corresponding author has registered an ORCID iD (0009-0006-0617-2462).

9. Please amend either the title on the online submission form (via Edit Submission) or the title in the manuscript so that they are identical.

**Reply:** We checked that the title in the manuscript was consistent with the title submitted online.

10. Please include captions for your Supporting Information files at the end of your manuscript, and update any in-text citations to match accordingly. Please see our Supporting Information guidelines for more information:

**Reply:** We include captions for our Supporting Information files at the end of our manuscript, and update any in-text citations to match accordingly. Please see the revised manuscript.

Special thanks to reviewers for your good comments and suggestions. We have tried our best to improve the manuscript, and here we did not list the changes but marked in red in revised paper. We appreciate for Reviewers and Editors warm work earnestly, and hope that the correction will meet with approval.
